# Current Research on Antioxidant, Anti-Inflammatory and Anti-Obesity Potential of Food Extracts

**DOI:** 10.3390/foods12051013

**Published:** 2023-02-27

**Authors:** Fernando Rivero-Pino, Maria C. Millán-Linares, Sergio Montserrat-de la Paz

**Affiliations:** Department of Medical Biochemistry, Molecular Biology, and Immunology, School of Medicine, Universidad de Sevilla, Av. Sanchez Pizjuan s/n, 41009 Seville, Spain

The immune-inflammatory, glucose homeostasis, and antioxidant response have a crucial role in the prevention of non-communicable chronic diseases, according to the World Health Organization (WHO). As people live longer in society, the lifestyle of human changes and the prevalence of age-related degenerative diseases and obesity is likewise rising quickly. One of the subfields of nutritional sciences, which covers the study of nutrients and other food ingredients with an emphasis on how they affect mammalian physiology, health, and behaviour, is nutritional biochemistry. Immunonutrition is a new and interdisciplinary field within Nutritional Biochemistry since it encompasses many elements of nutrition, immunity, inflammation, oxidation, and illnesses with an immunometabolic basis. One of the promising ingredients that are currently being assessed by scientists in terms of immunonutrition is food extracts. 

Food vegetable extracts are produced directly from fruits, vegetables, seeds, or roots of a plant and contain components that can perform a beneficial function in the body when ingested through food (fortified) or as a dietary supplement. These extracts can be used, for instance, as flavourings, natural dyes, stabilizers, or antioxidants. Over the past few decades, a thorough screening of dietary components has been carried out, and a wide spectrum of antioxidant, anti-inflammatory, and anti-obesity actions has been noted. These bioactivities exerted by food extracts could help promote a healthier lifestyle in humans by preventing and/or pre-treating many diseases, from diabetes or cardiovascular diseases to neurological diseases such as Alzheimer’s. In this regard, several ingredients have been reported as potential nutraceuticals from a wide variety of extracts, including hemp oil [1], artichoke by-products [2], red rice bran [3], chia [4], Fabaecea beans [5], mulberry branches [6], and cocoa [7]. These results indicate the potential of vegetable sources as health-promoting ingredients that can be used as food for humans. 

The production and consumption of extracts from vegetable sources satisfy the sustainable development goals (SDGs) established by Agenda 2030, including zero hunger, good health and well-being, responsible consumption and production, and climate action. It is well known that vegetable sources possess an environmentally friendly character, aside from the health benefits that their consumption has on human beings [1,2,3,4,5,6,7]. In addition, the use of by-products can also promote the circular economy, which is the ideal objective that will be used to define the food system in the near future [2,6]. In this regard, parts of botanicals which at the beginning are not used for any purpose can be revalued by applying processing techniques, releasing, or purifying bioactive compounds, such as alkaloids, flavonoids, and coumarins obtained from branches [6] or chlorogenic acid, naringin, and rutin [2], among other phenolic compounds, obtained from stems. Each part of the plant has a different composition of bioactive compounds, and the season in which these are collected can have an impact on the profile of bioactive molecules that the extract can have.

Different processes can be employed to extract bioactive ingredients, such as enzymatic hydrolysis [4,7], which can promote the release of peptides from native proteins using specific proteases that mimic the specificity of digestive proteases or not. Concerning conventional extraction, these include ethanol extraction [1,3,5], methanol extraction [6], and aqueous extraction [2]. Different solvents lead to the concentration of different components. In addition, some of the reports hereby included also take advantage of pre-treatments that are considered sustainable processing techniques, such as pulsed-electric fields [2] or ultrasound [5], which have been proved to promote the release of bioactive compounds compared to the direct extraction without pre-treatment and, thus, increasing the added-value of these extracts. These green extraction techniques have emerged to increase the extractability of valuable intracellular compounds from plant cell tissues with shorter extraction times, less energy, and solvent consumption while maintaining the integrity of the major labile extracted bioactive.

In the screening of several compounds, and as the first approach in choosing which compounds should be subjected to further analysis, in vitro/characterization studies can be carried out to establish which compound/extract could be more potent. Tungmunnithum et al. [5] indicated that *Vigna unguiculata* subsp. sesquipedalis populations (*n* = 50) showed low variability in their total phenolics (628.3 to 717.3 mg/100 g DW gallic acid equivalent), whereas the flavonoid and anthocyanin contents were highly variable (ranging from 786.9 to 1536.1 mg/100 g DW quercetin equivalent, and 13.4 to 41.6 mg/100 g DW cyanidin equivalent). The anti-obesity properties identified by in vitro analysis were reported for the mulberry branch extract, which was indicated to inhibit alpha-glucosidase (half maximal inhibitory concentration (IC_50_) of 1.90 ± 0.05 μg/mL), acetylcholinesterase and butyrylcholinesterase (IC_50_ of 179.47 ± 0.38 μg/mL and 101.82 ± 3.37 μg/mL) and to be antioxidant with scavenging DPPH and ABTS radicals.

Most of the analysis reported in the published papers in this Special Issue refers to cell-based assays [1,2,4,6], which help unravel the underlying mechanisms by which specific compounds exert bioactivities. Cell-based assays garner a lot of interest as a better compromise between in vivo investigations and in vitro chemical assays because both in vivo and in vitro assays have limitations. By measuring cytokines, (proinflammatory and anti-inflammatory), usually in macrophages, the beneficial effects on the immune response due to the specific compounds can be described and quantified. Chia (*Salvia hispanica* L.) seed peptides, which are considered a novel food in Europe, were reported to reduce reactive oxygen species and nitrite output and enhance the expression and release of anti-inflammatory cytokines [4]. Furthermore, the chia peptides reversed LPS-associated M1 polarization into M2, which could be related to a variety of human inflammatory diseases.

Animal studies precede human trials and are extremely useful in confirming the potential bioavailability and bioactivity of ingredients. In this Special Issue, in vivo anti-adipogenic, anti-hypertrophic, and anti-inflammatory activities of red rice bran extract were evaluated [3], highlighting the role of phenolics, flavonoids, anthocyanins, and proanthocyanidins in an obese mouse model. The extract was effective against high-fat diet-induced pathological changes in white adipose tissue via the down-regulation of genes involved in adipogenesis, lipogenesis, lipolysis, and inflammation after the consumption of 1 g/kg/day over 6 weeks [3]. Furthermore, the in vivo antihypertensive properties of cocoa peptides were reported, likely due to the inhibition of the angiotensin-converting enzyme [7]. In the investigation, the consumption of the cocoa extract, consisting of peptides, resulted in a decrease in systolic blood pressure by 5% and diastolic blood pressure by 7%. In addition, recently, the relevance of in silico analysis has gained interest within the research community, and this shows in the evaluation carried out by Coronado-Caceres et al. [7], who identified 26 cocoa peptides with alignment in the Blast^®^ analysis. The identification of specific peptides and the molecular docking analysis revealed that LSPGGAAV, TSVSGAGGPGAGR, and TLGNPAAAGPF could be the most potent sequences of the test item evaluated.

Some relevant results are reported in this Special Issue, for the first time, such as (i) the reprogramming and anti-inflammatory activity of phytol (obtained from hemp seeds (*Cannabis sativa* L.) for non-drug varieties, which are widely used as a source of medicines, foods, or fibres in human monocyte-macrophages [1]; (ii) the mechanisms by which the red rice bran extract attenuates adipogenesis and inflammation on white adipose tissues in high-fat diet-induced obese mice [3]; (iii) the application of the PEF-assisted extraction of phenolics from artichoke stems and the subsequent evaluation of the biological effects of these extracts using human cell lines [2]. These reports can be considered cutting-edge research in terms of the extraction, characterization, and evaluation of food extracts since together they report the use of ingredients considered as novel or from residues (not previously evaluated), in addition to the use of novel non-thermal extraction techniques (ultrasound or PEF) and making use of advanced bioactivity evaluation technologies such as cell cultures (of human cells) and animals.

The “Farm to Fork” Strategy aims to foster a circular economy; one of its goals is to lessen the agri-food industry’s negative environmental effects. It also aims to adopt measures that can boost the capacity of researchers to develop new sustainable food products and to promote access to nutritious food for all. These results, such as vegetable products and/or by-products, are employed to produce health-promoting ingredients that encompass and try to solve both the environmental problem and the development of diseases, which have been increasing in the human population recently due to lifestyle changes.

The research papers published in this Special Issue represent the potential of novel bioactive ingredients in the nutraceutical industry, addressing their biochemical, physiological, and molecular processes underlying mechanisms of action by different types of analysis. These reports offer an idea of how different food extracts that can be obtained from vegetable sources are adequate ingredients to be used as an antioxidant, anti-obesity, and/or anti-inflammatory nutraceutical based on their ability to modulate different physiological processes. However, the complexity of these extracts (considering the variety of compounds of different natures that are contained) makes it difficult to draw global conclusions on which compounds are more likely to exert certain bioactivity. For that purpose, some of the following studies also evaluate the potential of single compounds that can be identified as major compounds in the extracts.

Nonetheless, further research is required, by all interested parties (academia, industry, consumers, risk assessors), to ensure that these ingredients are feasible for use in the long-term in humans, modulating physiological status and health.
